# Estimation of battery SOC using a combined approach of temporal convolutional networks and Unscented Kalman Filter

**DOI:** 10.1371/journal.pone.0350331

**Published:** 2026-06-04

**Authors:** Huipin Lin, Xiaoying Chen, Lei Zhang, Zhengyi Bao, Sai Tang

**Affiliations:** 1 School of Electronics and Information, Hangzhou Dianzi University, Hangzhou, China; 2 Wenzhou Institute of Hangzhou Dianzi University, Wenzhou, China; 3 School of Electrical Engineering, Zhejiang University, Hangzhou, China; 4 School of Mechanical and Electrical Engineering, China Jiliang University, Hangzhou, China; UC: University of Calgary, CANADA

## Abstract

In recent years, power batteries have been widely used in electric vehicles, and the evaluation of state of charge (SOC) is an important parameter in battery management systems. Therefore, in this paper, we propose a time-series convolutional network that employs extended convolution and residual concatenation to efficiently process time-series data with parallelism and flexibility, and combines it with Unscented Kalman Filter (UKF) to further improve the accuracy and reduce the output fluctuation, so as to estimate the state of charge of lithium-ion batteries. We conducted experiments using the University of Maryland’s Dynamic Stress Test (DST), US06 test, and Federal Urban Driving Scheme (FUDS) datasets, and compared the proposed method with Convolutional Neural Networks (CNNs), Long and Short-Term Memory Networks (LSTMs), and Gated Recurrent Units (GRUs). Experimental results demonstrate that the proposed framework achieves superior estimation accuracy and robustness. Specifically, the proposed method achieves mean absolute error (MAE) values of 1.305%, 1.470%, and 1.015% under the DST, US06, and FUDS conditions, respectively, with an average Root Mean Square Error (RMSE) of 1.566% and a MAE below 1.263%. Compared with existing deep learning models, the proposed method reduces the SOC estimation error by approximately 7.6%–39.6% under different driving conditions. These results verify the effectiveness and robustness of the proposed hybrid SOC estimation framework.

## 1. Introduction

Rapid industrialization and urbanisation have exacerbated problems such as greenhouse gas emissions and fossil fuel consumption. In this context, the emergence of electric vehicles as an alternative to conventional gasoline vehicles is of great importance, mitigating pollutant emissions and energy constraints [1].

Lithium-ion batteries have wide applications in various fields such as electric vehicles, portable electronic devices and renewable energy storage systems. Accurate assessment of their remaining state of charge (SOC) is essential to ensure their reliability and safety. Especially in the field of electric vehicles, lithium-ion batteries have become the best battery technology and play a key role in accelerating the development of the electric vehicle industry [2]. Compared with other batteries used in electric vehicles, lithium-ion batteries have significant advantages: high energy density, high performance, long life cycle and low self-discharge rate [3]. SOC is a key indicator for evaluating the operating status of electric vehicles [4]. SOC indicates the remaining battery capacity, which is expressed as the ratio of the remaining battery capacity to the current maximum usable capacity [5]. However, estimating the SOC is a challenging task due to the nonlinear and dynamic properties of batteries and the influence of various factors such as temperature, current and aging, as well as the fact that batteries have hysteresis near the end of discharge [6,7].

### 1.1. Literature review

The most commonly used SOC estimation methods are the open-circuit voltage method, the ampere-time integration method, the model-based estimation method and the data-driven estimation method [8,9]. The open-circuit voltage method first establishes the relationship between open-circuit voltage and SOC based on the characteristic curve or model of the battery. The actual SOC is estimated by measuring the battery’s open-circuit voltage and matching it with the predetermined relationship curve [10]. The method is simple and highly accurate, but it can only be used when the vehicle is stationary and not in driving mode due to the hysteresis phenomenon inside the battery.

The ampere-time integration method calculates the amount of discharge for the time period by integrating the discharge current over time and combining it with the initial value for SOC estimation. The method is simple and efficient, but depends on the accuracy of the initial value and the influence of the measurement, and there will be cumulative errors [11]. Furthermore, the ampere-time integration method cannot account for changes in the internal state of the battery and the effects of internal resistance, polarization effects and temperature on the SOC estimation should also be considered in practical applications.

The model-based estimation method includes the establishment of a battery model and a model-based non-linear observer. Battery models mainly include electrochemical models [12], electrochemical impedance models and circuit models [13,14]. Combining a non-linear observer based on the model mainly involves passing inputs through the model outputs and applying algorithms for intelligently updating the model state as a way to dynamically estimate the battery state, commonly including Kalman filters, particle filters and H-infinity filters. Sepasi et al. proposed a model adaptive extended Kalman filtering-based SOC estimation for Li-ion, for overcoming battery ageing [15]. Ye et al. proposed an improved adaptive particle swarm filtering method that is extremely useful for eliminating errors due to battery degradation and initial SOC values [16]. An algorithm based on Thevenin equivalent circuit model and combined with extended Kalman filtering was first established by [17], which was shown to have higher estimation accuracy through validation. Meng et al. proposed an estimation method based on adaptive unscented Kalman filter (UKF) and a minimum support vector machine with high precision and applicability [18]. However, model-based estimation methods are often highly dependent on the establishment of the underlying battery model, and the internal operating conditions of the battery are extremely complex, making it impossible to establish a unified battery model so far.

As data volumes skyrocket, data-driven methods are gaining traction. Unlike other methods, they do not require complex modeling and calculations, and can directly estimate SOC by measuring battery data such as current, voltage, temperature, internal resistance, etc. Juan et al. proposed SOC estimation of high-capacity lithium iron-manganese phosphate batteries using a support vector machine (SVM) method [19]. Sahinoglu et al. proposed a machine learning-based approach for lithium-ion battery SOC estimation using a Gaussian process regression (GPR) framework to perform online SOC estimation, quantify uncertainty, and enable reliability assessment [20,21]. Neural networks and deep learning-based estimation methods are important approaches for SOC estimation as they are adaptive and self-learning and can build complex nonlinear models [22]. They consider the battery as a black box and estimate SOC by establishing a nonlinear relationship between SOC and input data (voltage, current, temperature) by training with a large amount of battery charge/discharge data [23]. Although this estimation method does not require the establishment of a specific battery model, it often requires a large amount of data, the quality and quantity of which determine the estimation performance of the method. In addition, the rapid development of graphics processing unit machine learning frameworks has rapidly advanced the level of data processing and the ability to train and build neural networks, providing technical support for the feasibility of this method [24,25]. Chaoui et al. designed a nonlinear autoregressive DDRN (NARX) structure with exogenous inputs [26]. This structure is used to estimate the SOC and health with improved computational intelligence and robustness.

Commonly used deep learning models include convolutional neural networks (CNNs) and recurrent neural networks (RNNs) [27–29]. The structure of convolutional neural networks (CNNs) can share local weights, allowing the networks to learn in parallel and handle high-dimensional data well. Fan et al. [30] proposed a battery capacity estimation method based on convolutional neural networks using relaxation voltages as inputs, and obtained good experimental results. However, CNN-based models mainly focus on local feature extraction and have limitations in modeling very long-term temporal dependencies in sequential data. To address time-series modeling problems, RNNs have been widely applied because they can capture temporal dependencies in sequential data. However, due to the recurrent structure of RNNs, gradient vanishing or gradient explosion may occur during the training process, which affects the learning efficiency and stability of the model [31,32]. To alleviate these issues, long short-term memory (LSTM) networks were proposed as an improved variant of RNNs. Yang et al. proposed an LSTM for battery state-of-charge estimation, demonstrating the superior performance of LSTM estimation [33]. A gated recurrent unit (GRU) is a simplified variant of LSTM that reduces computational complexity while retaining the ability to capture long-term dependencies. Yang et al. proposed a GRU-based RNN model to estimate the SOC of a battery by measuring current, voltage, and temperature [34]. In addition, hybrid methods combining neural networks and filtering techniques have also been investigated to further improve estimation accuracy. For example, Yang et al. combined an LSTM network with a UKF to reduce estimation noise, while Tian et al. proposed a hybrid method integrating LSTM with adaptive culture Kalman filtering (ACKF), which significantly improves estimation accuracy and generalization performance compared with using LSTM alone. Nevertheless, RNN-based methods still suffer from inherent structural limitations due to their sequential computation mechanism, which makes them difficult to train efficiently on long sequences and may still lead to gradient instability. Recently, Temporal Convolutional Networks (TCNs) have been introduced as an effective alternative for time-series modeling. Compared with recurrent architectures, TCN employs dilated causal convolutions and residual connections, enabling the network to obtain a significantly larger receptive field while maintaining stable training. This structural characteristic allows the model to capture long-term temporal dependencies more effectively. For battery SOC estimation, such long-term dependencies are closely related to electrochemical recovery effects, polarization dynamics, and aging processes that evolve gradually during battery operation. The dilated convolution mechanism of TCN enables the model to incorporate historical information over a wider temporal range without increasing computational complexity, making it particularly suitable for capturing the slow dynamic behavior of lithium-ion batteries. Liu et al. proposed a TCN-based SOC estimation method combined with transfer learning, demonstrating that TCN can effectively model battery time-series data [35]. In general, TCN has shown strong capability in handling complex time-series prediction tasks [36]. However, real-world battery measurements often contain significant noise due to sensor inaccuracies and operational disturbances, which may degrade model performance. To address this issue, researchers have explored various strategies to improve robustness under noisy conditions [37]. For instance, Fan et al. [38] proposed a co-learning framework combining supervised learning and self-supervised learning to estimate battery capacity under noisy data conditions.

In this paper, a TCN is developed for real-time SOC estimation. Considering that the measurements collected from battery sensors often contain noise and fluctuations, a UKF is integrated at the backend of the network to perform post-processing on the predicted SOC values, thereby forming a hybrid TCN-UKF estimation framework. In this framework, SOC estimation is formulated as a time-series learning problem. The battery measurement data are first processed and fed into the designed TCN model, where temporal features are extracted to generate preliminary SOC predictions. Subsequently, the UKF is applied to perform nonlinear state filtering and measurement correction, which effectively suppresses output fluctuations caused by sensor noise and measurement uncertainty. Compared with recurrent neural network based hybrid approaches such as LSTM-UKF, the proposed TCN-UKF framework offers several theoretical advantages for SOC estimation. Specifically, the TCN employs dilated causal convolutions and residual connections, which enable the model to capture long-term temporal dependencies while maintaining efficient parallel computation. Unlike LSTM networks that rely on sequential recurrence, the convolutional structure of TCN allows more stable gradient propagation and avoids the long-term dependency degradation commonly observed in recurrent architectures. In this hybrid architecture, the TCN is responsible for extracting nonlinear temporal features from battery measurements, while the UKF performs dynamic filtering and error correction on the predicted SOC. As a result, the proposed framework can effectively enhance estimation stability and robustness under complex driving conditions. The main contributions of this work are:

- We propose a TCN-based model for SOC estimation, which eliminates the need for complex battery modeling and intricate parameter tuning. This significantly reduces the overall estimation complexity, thereby simplifying both model construction and data processing.
- To enhance estimation accuracy, the UKF is integrated to refine the network’s predictions. By fully accounting for the battery’s inherent nonlinearities, the filtering process produces smoother SOC estimation results with reduced error, thus improving both accuracy and robustness and broadening the practical applicability of the proposed method.
- The impact of varying initial conditions on SOC estimation is also investigated. Experimental results demonstrate the model’s performance under diverse operating conditions, including variations in load profiles, ambient temperature, and initial SOC levels. These results confirm the proposed model’s strong adaptability and robustness in real-world battery management scenarios.
- In addition, the system model proposed in this study is compared with several widely adopted SOC estimation approaches, including CNN, LSTM, GRU, and standalone TCN models. Using publicly available datasets for validation, experimental results demonstrate that the proposed method effectively reduces the initial value estimation error, significantly enhances estimation accuracy, and improves the model’s generalization capability.

### 1.2. Organization of this paper

The rest of the paper is organized as follows. Section 2 details the structure of TCN and UKF, as well as an overview of the overall network framework. Section 3 summarizes the data sources and normalization process. Section 4 is used to conduct comparative experiments and document the results. Finally, Section 5 summarizes the whole paper.

## 2. Methodologies

### 2.1. TCN

**Fig 1:**
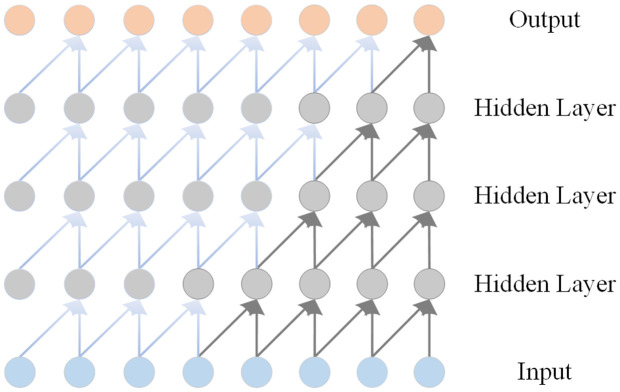
Causal convolution block with four layers with kernel size 2.

TCN is a neural network model for processing time series data, which can be regarded as a special kind of CNN. Compared with RNNs, which need to process input data sequentially step by step, TCNs can be processed in parallel, which greatly improves the training and inference efficiency of the network, and the size of the receptive field can be flexibly adjusted by adjusting the parameters of the number of layers, convolutional kernel size, and scaling factor to meet different task requirements. Compared with RNNs, TCNs have advantages in the problems of gradient vanishing and gradient explosion. These advantages are mainly attributed to the unique network architecture of TCN, which can not only handle time series data, but also overcome some limitations of traditional convolutional networks. TCN adopts the architecture of one-dimensional full convolutional network (1D FCN), and the length of each hidden layer is equal to the length of the input layer. Zero padding is used to keep the lengths between layers equal. The causal convolutional module of this architecture consists of four convolutional layers, as shown in Fig 1, and the convolutional kernel size of each layer is 2. Causal convolution is characterized by not sharing the parameters of different time steps because the output at each moment depends only on the current moment and previous inputs and cannot see future data, so it is a strictly time-constrained model. In addition, TCNs share convolutional kernels in the same layer, which reduces memory requirements.

**Fig 2:**
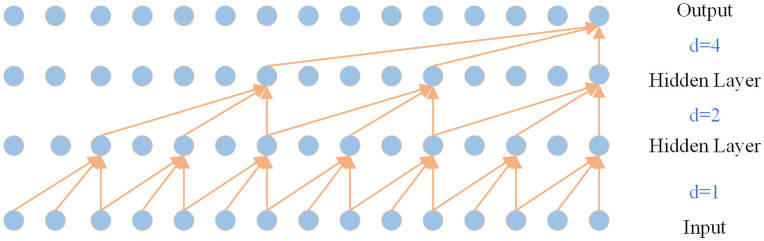
Dilated causal convolution block with three layers.

However, increasing the feeling field through causal convolution increases the model complexity. To address this issue, TCN introduces inflationary convolution. With inflationary convolution, the input can be sampled at intervals, and the sampling rate is determined by the parameter d. The value of d indicates how many points are sampled as input, and the value of d tends to increase as the number of layers increases. With extended convolution, the effective receptive field increases exponentially with the number of layers, so you can get a larger receptive field with fewer layers. Fig 2 shows the extended convolution module in TCN.

**Fig 3:**
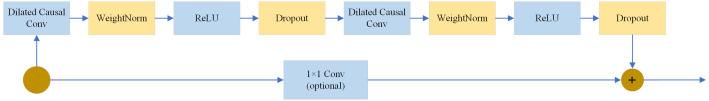
TCN residual block.

Residual connectivity allows the network to transfer information between different layers, mitigates the problem of vanishing gradients, and can effectively train the optimization network to improve its learning and performance capabilities. In TCN, each residual block consists of two causal convolution layers with zero padding, weight normalization, ReLU activation and dropout. If the number of input channels differs from the number of output channels in the extended causal convolution, a 1x1 convolutional layer can be added to ensure that the remaining connections are valid. The design of the residual block not only allows the network to efficiently transfer information between layers, but also ensures that inputs and outputs are consistent. Fig 3 illustrates the structure of the residual block in TCN.

Specifically, assume that the input sequence is denoted as $X=\left[x_{1},x_{2},...,x_{T}\right]$, where $x_{t}\in R^{3}$ denotes the voltage, current, and temperature at the *t*-th time step. each layer of the TCN performs the expansion one-dimensional convolution operation as follows:


$$y_{t}^{\left(l\right)}=\sum_{k=0}^{K-1}w_{k}^{\left(l\right)}\cdot x_{k-d\cdot k}^{\left(l-1\right)}+b^{\left(l\right)}$$ (1)

where $y_{t}^{\left(l\right)}$ represents the output of the *l*-th layer at time step t. The *x*^(*l*−1)^ is the output of the previous *l*ayer. $w_{k}^{\left(l\right)}$ is the *k*-th weight of the convolution kernel and *k* is the convolution kernel size. *d* is the dilation factor. *b*^(*l*)^ is the bias term of the layer. Each convolutional layer is followed by a nonlinear activation layer and a normalization operation:


$${\tilde{y}}_{t}^{\left(l\right)}=Dropout\left(\sigma \left(LN\left(y_{t}^{\left(l\right)}\right)\right)\right)$$ (2)

where $y_{t}^{\left(l\right)}$ represents the output of the *l*-th where, σ(·) represents the activation function sigmoid. LN(·) stands for normalization. Fina*l*ly, after stacked TCN processing, the final output vector is the predicted output of the TCN.

### 2.2. UKF

The UKF employs the unscented transform (UT) within the standard Kalman filtering framework to handle nonlinear system dynamics. Unlike the Extended Kalman Filter, the UKF avoids explicit linearization of the system model, yielding more accurate state estimates. Furthermore, the UKF scales effectively to high-dimensional state spaces, making it well suited for large-scale nonlinear systems. In this section, we first introduce the implementation process of the UKF, followed by a detailed explanation of the specific implementation process for the TCN-UKF described in this paper. The specifics are as follows:

#### 2.2.1. UKF implementation process.

(a) Set the initial state estimate ${\hat{x}}_{0}$ and the covariance *P*_0_.


$${\hat{x}}_{0}=E\left(x_{0}\right)$$ (3)

$$P_{0}=E\left[\left(x_{0}-{\hat{x}}_{0}\right){\left(x_{0}-{\hat{x}}_{0}\right)}^{T}\right]$$ (4)

(b) Obtain a set of sampling points and corresponding weights.


$${x^{i}}_{\left(k-1\right)}={\hat{x}}_{\left(k-1\right)},i=0$$ (5)

$$x_{\left(k-1\right)}^{i}={\hat{x}}_{\left(k-1\right)}+{\left(\sqrt{(n+\lambda )P_{\left(k-1\right)}}\right)}_{i},\;1\leq i\leq n$$ (6)

$$x_{\left(k-1\right)}^{i}={\hat{x}}_{\left(k-1\right)}-{\left(\sqrt{(n+\lambda )P_{\left(k-1\right)}}\right)}_{i-n},\;n+1\leq i\leq 2n$$ (7)

(c) Compute one-step predictions for sigma point sets.


$$x_{k|k-1}^{i}=f\left(x_{k-1}^{i},u_{k-1}\right)$$ (8)

(d) Predictions of a set of sigma points are utilized and weighted means are computed to obtain state variable predictions and covariances for further updating.


$${\hat{x}}_{\left(k|k-1\right)}=\sum_{i=0}^{2n}w_{i}^{m}x_{\left(k|k-1\right)}^{i}$$ (9)

$$P_{\left(x,k|k-1\right)}=\sum_{i=0}^{2n}w_{i}^{c}(x_{\left(k|k-1\right)}^{i}-{\hat{x}}_{\left(k|k-1\right)})(x_{\left(k|k-1\right)}^{i}-{\hat{x}}_{\left(k|k-1\right)}{)}^{T}+Q_{k}$$ (10)

(e) Substitute the predicted Sigma point set into the observation equation to obtain a one-step prediction of the observation.


$$Z_{\left(k|k-1\right)}^{i}=h\left(x_{\left(k|k-1\right)}^{i}\right)$$ (11)

(f) The observed predictions of the Sigma point set are obtained and the mean and covariance of the system predictions are obtained by weighted summation.


$${\hat{Z}}_{\left(k|k-1\right)}=\sum_{i=0}^{2n}w_{i}^{m}Z_{\left(k|k-1\right)}^{i}$$ (12)

$$P_{zz,k}=\sum_{i=0}^{2n}w_{i}^{c}(Z_{\left(k|k-1\right)}^{i}-{\hat{Z}}_{\left(k|k-1\right)})(Z_{\left(k|k-1\right)}^{i}-{\hat{Z}}_{\left(k|k-1\right)}{)}^{T}+R_{k}$$ (13)

$$P_{xz,k}=\sum_{i=0}^{2n}w_{i}^{c}(x_{\left(k|k-1\right)}^{i}-{\hat{x}}_{\left(k|k-1\right)})(Z_{\left(k|k-1\right)}^{i}-{\hat{Z}}_{\left(k|k-1\right)}{)}^{T}$$ (14)

(g) Calculating Kalman Gain, then update System state and covariance.


$$K_{k}=P_{xz,k}P_{zz,k}^{-1}$$ (15)

$${\hat{x}}_{\left(k|k\right)}={\hat{x}}_{\left(k|k-1\right)}+K_{k}({\hat{Z}}_{k}-{\hat{Z}}_{\left(k|k-1\right)})$$ (16)

$$P_{x,k|k}=P_{x,k|k-1}-K_{k}P_{zz,k}{K_{k}}^{T}$$ (17)

The equations of state and observation equations for SOC estimation are given by the ampere-time integration method. where *I* is the current, *T* is the sampling period, *C*_*n*_ is the nominal capacity of the battery, *SOC*_*k*_ is the ampere-hour integration method, and *TCN*_*k*_ is the output SOC of the TCN network at time step *k*.


$$SOC_{k}=f(SOC_{k-1})=SOC_{k-1}-\frac{I_{k-1}T}{C_{n}}+\omega_{k}$$ (18)

$$z_{k}≜TCN_{k}=h(SOC_{k})+\nu_{k}=SOC_{k}+\nu_{k}$$ (19)

The process noise covariance *Q* and the measurement noise covariance *R* are important parameters that affect the convergence and stability of the UKF. In this study, these parameters are determined based on empirical tuning combined with preliminary validation experiments. Specifically, *Q* reflects the uncertainty of the system state transition model derived from the coulomb-counting equation, while *R* represents the uncertainty of the SOC pseudo-measurement generated by the TCN network. In practice, small values of *Q* are adopted to ensure smooth SOC state evolution, whereas *R* is selected according to the statistical characteristics of the TCN estimation error obtained from the validation dataset. Through multiple preliminary experiments, the values of *Q* and *R* are adjusted to achieve stable filter convergence and optimal estimation accuracy. This empirical tuning strategy is widely used in nonlinear filtering applications when the exact noise statistics are difficult to obtain.

#### 2.2.2. TCN-to-UKF measurement mapping and update procedure.

To explicitly couple the TCN and UKF in an end-to-end manner, we treat the TCN output as a *pseudo-measurement* of SOC and fuse it with the UKF prediction at each time step.

Let the UKF state be defined as:


$${𝐱}_{k}=[\;{\text{SOC}}_{k}\;{]}^{⊤}$$ (20)

and the input vector consists of measured battery signals (e.g., voltage, current, and temperature) denoted by **u**_*k*_. Given a sliding window of measurements ${𝐔}_{k}=\{{𝐮}_{k-L+1},\ldots ,{𝐮}_{k}\}$, note that the state transition from *k* − 1 to *k* uses the input *u*_*k*−1_ (e.g., *I*_*k*−1_ in coulomb counting), while the TCN takes a measurement window up to time *k* to produce the pseudo-measurement ${\hat{z}}_{k}$ for the UKF correction.


$${\hat{z}}_{k}=f_{\text{TCN}}({𝐔}_{k})$$ (21)

which is used as the UKF measurement at time step *k*. Accordingly, the UKF measurement model is defined as:


$$z_{k}=h({𝐱}_{k})+\nu_{k},\;h({𝐱}_{k})={\text{SOC}}_{k}$$ (22)

where $\nu_{k}\sim 𝒩(0,R)$ represents the measurement noise capturing the uncertainty of the TCN output.

**Time update:** The UKF propagates sigma points $\left\{\chi_{k-1}^{\left(i\right)}\right\}$ through the state transition function g(·):


$${𝐱}_{k}^{-}=\sum_{i}W_{m}^{\left(i\right)}\;g(\chi_{k-1}^{\left(i\right)},{𝐮}_{k-1}),\;{𝐏}_{k}^{-}=\sum_{i}W_{c}^{\left(i\right)}(g(\chi_{k-1}^{\left(i\right)},{𝐮}_{k-1})-{𝐱}_{k}^{-})(\cdot {)}^{⊤}+𝐐$$ (23)

where **Q** is the process noise covariance.

**Measurement update using the TCN output:** The predicted sigma points are mapped into the measurement space using h(·):


$$\zeta_{k}^{\left(i\right)}=h(\chi_{k}^{\left(i\right)})$$ (24)

and the predicted measurement mean and covariances are computed as:


$${\hat{z}}_{k}^{-}=\sum_{i}W_{m}^{\left(i\right)}\zeta_{k}^{\left(i\right)},\;{𝐒}_{k}=\sum_{i}W_{c}^{\left(i\right)}(\zeta_{k}^{\left(i\right)}-{\hat{z}}_{k}^{-})(\zeta_{k}^{\left(i\right)}-{\hat{z}}_{k}^{-}{)}^{⊤}+𝐑$$ (25)

$${𝐏}_{xz,k}=\sum_{i}W_{c}^{\left(i\right)}(\chi_{k}^{\left(i\right)}-{𝐱}_{k}^{-})(\zeta_{k}^{\left(i\right)}-{\hat{z}}_{k}^{-}{)}^{⊤}$$ (26)

Then, the Kalman gain and the posterior estimate are updated by:


$${𝐊}_{k}={𝐏}_{xz,k}{𝐒}_{k}^{-1},\;{𝐱}_{k}={𝐱}_{k}^{-}+{𝐊}_{k}({\hat{z}}_{k}-{\hat{z}}_{k}^{-}),\;{𝐏}_{k}={𝐏}_{k}^{-}-{𝐊}_{k}{𝐒}_{k}{𝐊}_{k}^{⊤}$$ (27)

Therefore, at each time step *k*, the TCN provides a data-driven SOC pseudo-measurement ${\hat{z}}_{k}$, and the UKF performs a principled Bayesian correction by weighting the predicted SOC and the TCN pseudo-measurement according to **Q** and **R**, which effectively suppresses output fluctuations and improves robustness under noisy operating conditions.

### 2.3. Overall network framework

**Fig 4:**
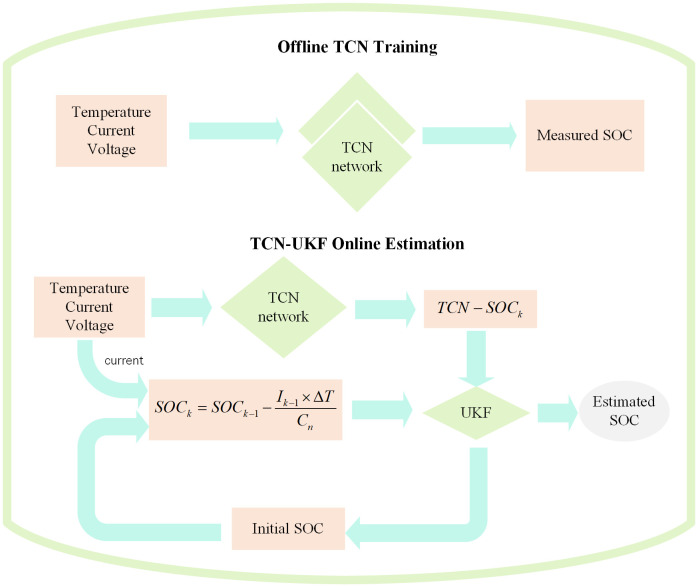
SOC estimation framework combining the TCN and UKF.

The system framework designed in this paper is shown in Fig 4 below and is divided into two phases, offline training and online testing. First, in the offline phase, battery measurements (e.g., voltage, current, and temperature) are used to train the TCN network and noise is filtered out by UKF to improve the estimation accuracy.

The TCN layer adopts a convolutional kernel size of 3 with 64 filters and dilation factors of 1, 2, 4, and 8, which enables the network to capture temporal dependencies at different time scales. To prevent overfitting and stabilize the training process, a dropout rate of 0.2 is applied. The activation function is set to sigmoid, and the final SOC estimation is obtained through a fully connected layer. The hyperparameters used in this study were determined through two steps. First, the feasible ranges of the hyperparameters were selected based on previous studies and empirical experience in battery state estimation tasks. Then, several combinations of kernel sizes, filter numbers, and dropout rates were evaluated through preliminary tuning experiments using the validation dataset to analyze their influence on SOC estimation accuracy and model generalization. The experimental results indicate that the above configuration provides a good balance between estimation accuracy and computational efficiency. For network training, the Adam optimizer is adopted with a learning rate of 0.0001. The batch size and number of epochs are set to 16 and 250, respectively. Since the differences between repeated experiments are relatively small, the average value of five independent runs is used as the final output to ensure the stability and reliability of the results.

The performance of the proposed network is evaluated using mean absolute error (MAE) and root mean square error (RMSE), where MAE proves the accuracy of the estimation and RMSE proves the robustness of the estimation. This is expressed by the following equation.


$$MAE=\frac{1}{n}\sum_{i=1}^{n}\left|y_{{\;}_{i}}-{\hat{y}}_{i}\right|$$ (28)

$$RMSE=\sqrt{\frac{1}{n}\sum_{i=1}^{n}{\left(y_{{\;}_{i}}-{\hat{y}}_{i}\right)}^{2}}$$ (29)

## 3. Datasets

Three dynamic load models were used to simulate different discharge modes of the EV battery: the Dynamic Stress Test (DST), the US06 test and the Federal Urban Driving Scheme (FUDS). The FUDS model focuses on EV battery use under urban driving conditions, while the US06 model addresses highway driving conditions. Data were obtained from the University of Maryland (CALCE) Advanced Life Cycle using 18650 LiNiMnCoO2 lithium graphite batteries, with key parameters shown in Table 1.

**Table 1:** Lithium battery parameters.

Type	Capacity Rating	Weight	Nominal Voltage	Nominal Capacity
18650-20R	2000mAh	45.0g	3.6V	2.0Ah

**Fig 5:**
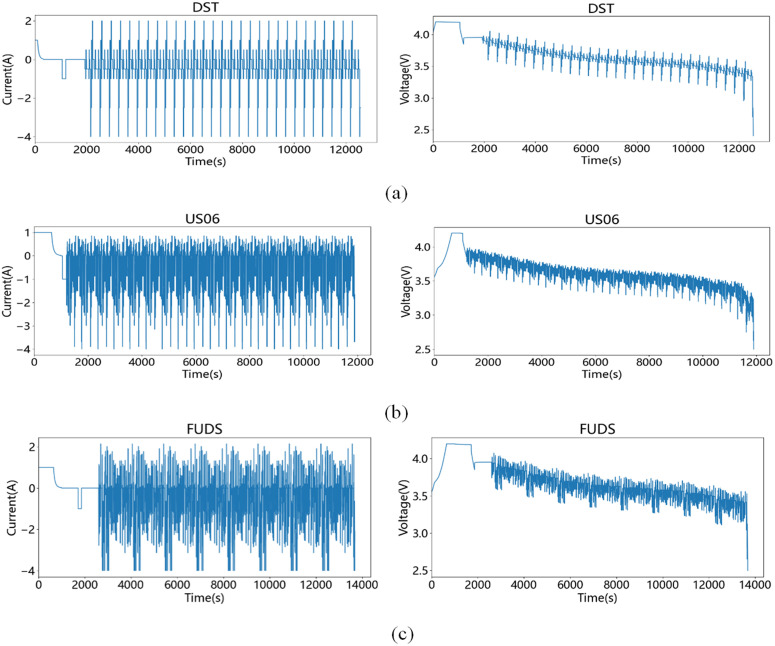
25°C battery charge/discharge curves: (a) DST condition; (b) US06 condition; (c) FUDS condition.

To evaluate the effectiveness of the proposed method, experiments are conducted using three operating conditions, namely US06, DST, and FUDS. In the experiments, the data from one operating condition is used as the training set, while partial data from the other two operating conditions are used as the test set. This design allows the model to be trained under one driving condition and evaluated under different unseen conditions, thereby assessing its cross-condition generalization capability. The experiments are conducted on a Windows 10 platform using the TensorFlow–Keras framework with Python 3.8, and MATLAB is employed to implement the UKF process. In this study, the training and testing datasets are strictly separated at both the battery level and the operating-condition level. Specifically, the batteries used for training and testing are completely different, and the corresponding operating conditions are also distinct. Therefore, the dataset partition follows an inter-cell splitting strategy rather than an intra-cell temporal segmentation. In other words, data from the same battery or the same discharge cycle never appear simultaneously in both the training and testing sets. This design effectively avoids potential data leakage and enables a more reliable evaluation of the generalization capability of the proposed method under unseen operating conditions. Fig 5 shows the current and voltage profiles of the battery during the testing process under the three operating conditions.

## 4. Results and discussion

### 4.1. Comparison with other methods

To ensure the fairness of the comparison among different deep learning models, all baseline methods (CNN, LSTM, GRU, and TCN) were implemented under the same experimental environment and trained using identical training settings. Specifically, the Adam optimizer was adopted with a learning rate of 0.0001, the batch size was set to 16, and the number of training epochs was fixed at 250 for all models. In addition, the architectures of the baseline models were selected based on commonly used configurations in previous battery SOC estimation studies to maintain comparable model capacities. The detailed hyperparameter settings of each model are summarized in Table 2. This unified experimental setup ensures that the performance differences mainly originate from the model structures rather than inconsistent parameter settings.

**Table 2:** Hyperparameter settings of all models used in the experiments.

Hyperparameter	CNN	LSTM	GRU	TCN	TCN-UKF
Kernel size	3	–	–	3	3
Dilation factors	–	–	–	1, 2, 4, 8	1, 2, 4, 8
Filters / Hidden units	32, 64, 128	64	64	64	64
Dropout	–	0.2	0.2	0.2	0.2
Learning rate	0.0001	0.0001	0.001	0.0001	0.0001
Batch size	16	16	16	16	16

**Fig 6:**
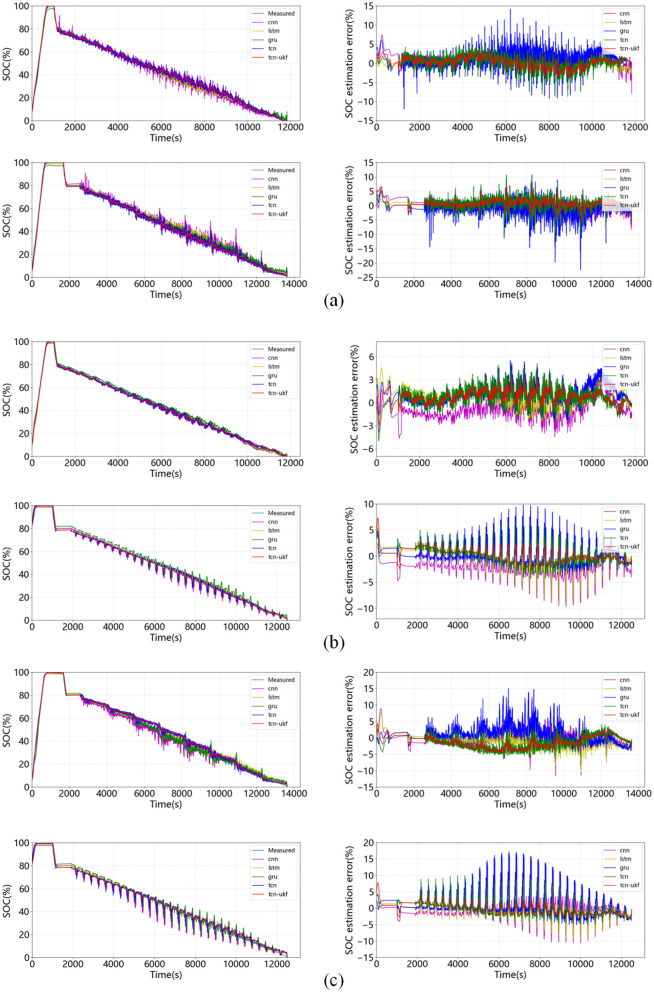
SOC estimates and errors for different operating conditions at 25°C: (a) DST condition, US06 test (top), FUDS test (bottom); (b) FUDS condition, US06 test (top), DST test (bottom); (c) US06 condition, FUDS test (top), DST test (bottom).

Under the three working conditions, TCN-UKF is compared with CNN, LSTM, GRU and the standalone TCN model, and the results are shown in Fig 6. The average RMSE of CNN reaches 2.36%, which is poor due to its limitation in processing the temporal information. LSTM has a better ability to process the information, and it has an average RMSE of 1.965%, but it cannot be processed in parallel. GRU has a simpler network structure than LSTM and it has a poorer average RMSE of 2.298%, TCN has the best results with an average RMSE of 1.752% but fails to achieve the best results due to the fact that it does not deal with the output fluctuations, whereas the proposed TCN-UKF method obtains the best results with an average RMSE of 1.565%. Table 3 records the estimation error for each training test set during the experiment at room temperature.

**Table 3:** SOC errors at room temperature.

Model	Train Data	Test Data	MAE(%)	RMSE(%)
CNN	DST	US06	1.714	2.285
		FUDS	1.759	2.587
	FUDS	US06	1.121	1.519
		DST	1.316	1.644
	US06	FUDS	1.850	2.570
		DST	2.272	3.580
LSTM	DST	US06	1.463	1.913
		FUDS	1.733	2.217
	FUDS	US06	1.112	1.370
		DST	1.337	1.712
	US06	FUDS	1.681	2.231
		DST	1.719	2.347
GRU	DST	US06	1.407	1.813
		FUDS	1.816	2.351
	FUDS	US06	1.293	1.524
		DST	2.341	2.790
	US06	FUDS	1.736	2.401
		DST	1.979	2.911
TCN	DST	US06	1.192	1.522
		FUDS	1.342	1.858
	FUDS	US06	1.025	1.308
		DST	1.267	1.593
	US06	FUDS	1.840	2.274
		DST	1.630	1.957
TCN-UKF	DST	US06	1.080	1.331
		FUDS	1.170	1.674
	FUDS	US06	0.950	1.139
		DST	1.110	1.371
	US06	FUDS	1.770	2.144
		DST	1.500	1.738

Based on the experimental results, it is obvious that the maximum error of the TCN-UKF algorithm for SOC estimation is around 5%, which is lower than that of other network prediction models, and the proposed algorithm outperforms the CNN, LSTM, GRU, and the TCN network alone in all three working conditions. It provides better estimation results with higher accuracy and smaller error. It can be concluded that the TCN-UKF algorithm is more efficient and robust than the TCN alone.

### 4.2. Estimation results of SOC at different temperatures

Temperature has an impact on the internal chemical state of the battery, which affects the battery SOC estimation, and when the battery is discharged, the internal temperature directly affects the rate of discharge and the battery capacity. Table 4 shows the SOC estimation errors at 0°C, 25°C, and 45°C using the TCN network as well as the TCN-UKF model with US06, FUDS, and DST as the conditions tested. It can be seen that the temperature has a significant effect on the SOC estimation. Therefore, investigating the performance of TCN-UKF for SOC estimation at different temperatures can help to fulfill practical applications.

**Table 4:** SOC errors at different temperatures.

Models	Test Data	Temperature(℃)	MAE(%)	RMSE(%)
TCN	US06	45	1.429	1.831
		25	1.192	1.522
		0	1.158	1.524
	FUDS	45	1.549	1.986
		25	1.342	1.858
		0	1.260	1.766
	DST	45	0.936	1.187
		25	1.267	1.593
		0	1.372	1.732
TCN-UKF	US06	45	1.290	1.740
		25	1.080	1.330
		0	1.020	1.310
	FUDS	45	1.420	1.870
		25	1.170	1.670
		0	1.080	1.490
	DST	45	0.820	1.030
		25	1.110	1.370
		0	1.180	1.460

**Fig 7:**
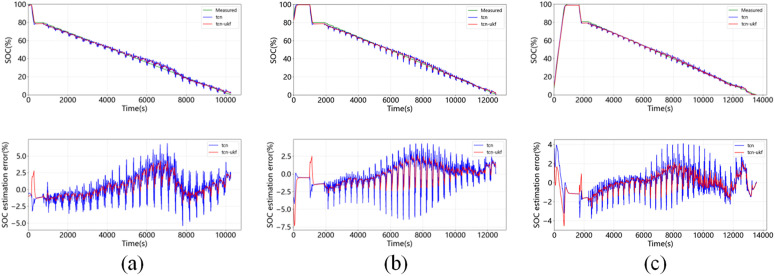
SOC estimation and error at different temperatures when DST is used as a test set: (a) 0℃; (b) 25℃; (c) 45℃.

**Fig 8:**
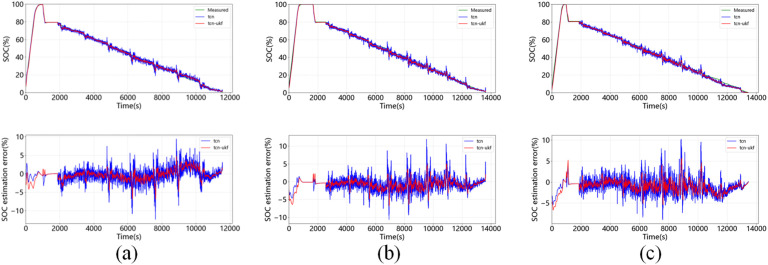
SOC estimation and error at different temperatures for FUDS as a test set: (a) 0℃; (b) 25℃; (c) 45℃.

**Fig 9:**
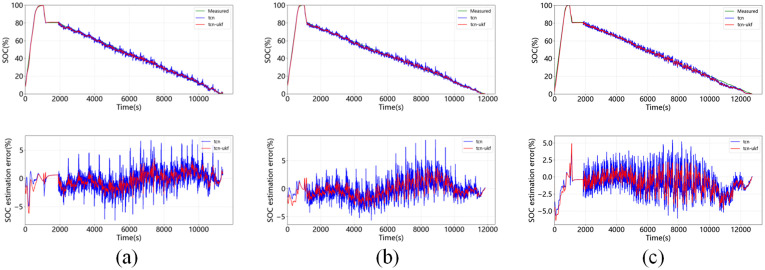
SOC estimation and error at different temperatures for US06 as a test set: (a) 0°C; (b) 25°C; (c) 45°C.

Fig 7 shows the estimation results of SOC at 0°C, 25°C, 45°C when DST is used as the test set. It shows that the error of the TCN-UKF model is less than that of the TCN network alone at all temperatures. Fig 8 shows the estimation results of SOC at 0°C, 25°C, 45°C with FUDS as a test set, showing that the maximum error of TCN-UKF model at different temperatures is not more than 5%, while the maximum error of normal TCN network is around 10%, and the proposed model outperforms the simple TCN estimation. Fig 9 shows the estimation results of SOC at 0°C, 25°C, 45°C using US06 as the test set. The maximum error of both models does not exceed 5%, but from the table, it can be seen that the estimation error of the TCN-UKF is significantly smaller than that of the TCN network. Therefore, embedding the UKF makes the estimation performance of the TCN network improved, and improves the robustness and accuracy of the model in predicting the SOC.

### 4.3. SOC estimation structure for different initial power levels

**Fig 10:**
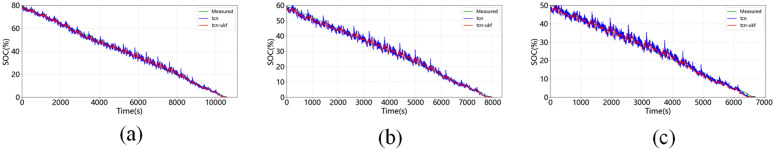
SOC estimation at different power levels: (a) 80%; (b) 60%; (c) 50%.

In practice, the initial power of the battery affects the initial voltage and current, and hence the SOC value, and the accuracy of battery SOC estimation changes when the initial discharge rate is high or low. It is extremely important that the model provides good estimation performance for different initial powers, as the SOC estimation error of the battery may increase if the initial power is too high or too low. Therefore, in order to investigate the accuracy of SOC estimation when the initial power is varied, an experiment was conducted at room temperature with DST as the training set and US06 as the test set, with initial power levels set to 80%, 60% and 50% respectively, to verify the estimation effectiveness of the model and compare it with TCN. The results are shown in Fig 10.

Experiments show that the TCN network accurately estimates SOC at 80%, 60% and 50% initial power, with an RMSE of 1.574% at 80% initial power, 1.679% at 60% initial power and 1.588% at 50% initial power. Fig 10 and the data show that the TCN-UKF model reduces the impact of different initial power on the SOC estimation error, filtering by UKF reduces the estimation fluctuations, the model prediction is more accurate, and through TCN-UKF, the SOC values under different initial power conditions are It can be seen that accurate estimation is possible and meets practical needs.

### 4.4. Time consumption and computational complexity analysis

#### Time consumption.

To quantitatively evaluate the computational efficiency of the proposed method, time consumption experiments were conducted on CNN, LSTM, GRU, TCN, and the proposed TCN-UKF framework. All models were trained under the same experimental settings to ensure a fair comparison. For the dataset splitting strategy described above, multiple experiments were conducted and the average computational time was reported. The training time and single-step inference time of different models are summarized in Table 5.

**Table 5:** Comparison of computational efficiency under different operating conditions.

Test Data	Model	Training Time (s)	Testing Time (s)
DST	CNN	15.78	20.31
	LSTM	69.06	83.84
	GRU	50.37	63.15
	TCN	27.92	31.92
	TCN-UKF	35.33	40.03
FUDS	CNN	19.73	22.28
	LSTM	75.62	83.79
	GRU	55.91	63.07
	TCN	33.77	37.87
	TCN-UKF	39.16	42.98
US06	CNN	14.54	27.35
	LSTM	61.67	85.92
	GRU	50.11	63.21
	TCN	21.47	29.96
	TCN-UKF	31.52	38.05

It can be observed that recurrent models such as LSTM and GRU require longer training time due to their sequential computation structure. In contrast, the TCN-based architecture benefits from convolutional parallelism, resulting in reduced training time. Although the proposed TCN-UKF framework introduces an additional filtering step, the increase in computational cost is limited. Therefore, the proposed method maintains efficient inference while improving estimation robustness.

#### Computational complexity analysis.

To provide a theoretical explanation for the observed computational efficiency, the computational complexity of different models is further analyzed. Let *T* denote the input sequence length, *d* the hidden dimension or number of channels, *k* the convolution kernel size, *L* the number of network layers, and *n* the state dimension of the UKF. The complexity of each model is summarized in Table 6.

**Table 6:** Theoretical computational complexity comparison of different models.

Model	Complexity	Reason
CNN	O(L·T·k·d)	Parallel; local receptive field.
LSTM	$O(L\cdot T\cdot d^{2})$	Sequential recurrence.
GRU	$O(L\cdot T\cdot d^{2})$	Lower-cost recurrent model.
TCN	O(L·T·k·d)	Dilated convolution.
TCN-UKF	$O(L\cdot T\cdot k\cdot d)+O(T\cdot n^{3})$	TCN + UKF overhead.

RNNs such as LSTM and GRU perform sequential computations, where each time step depends on the previous hidden state. Therefore, their time complexity is approximately $O(L\cdot T\cdot d^{2})$, which limits parallelization and leads to higher computational cost, especially for long sequences. In contrast, convolution-based models such as CNN and TCN can be computed in parallel across time steps. The complexity of TCN is approximately O(L·T·k·d). By leveraging dilated convolutions, TCN can significantly enlarge the receptive field without increasing the computational complexity, enabling efficient long-range temporal dependency modeling. For the proposed TCN–UKF framework, the additional computational overhead mainly arises from the UKF filtering process. The complexity of UKF is approximately $O(T\cdot n^{3})$, where *n* is the state dimension. Since the SOC estimation problem typically involves a low-dimensional state, the additional cost introduced by UKF remains limited in practice. Overall, the proposed TCN–UKF method achieves a favorable trade-off between computational complexity and estimation accuracy. This theoretical analysis is consistent with the experimental results in Table 5, where TCN-based models demonstrate superior efficiency compared to recurrent models, while the additional cost introduced by UKF remains acceptable for practical applications.

## 5. Conclusion

This paper proposes a hybrid battery SOC estimation framework that combines a TCN with the UKF. In the proposed approach, voltage, current, and temperature measurements are first processed by the TCN to obtain preliminary SOC estimates, and the UKF is subsequently employed to suppress noise and refine the estimation results. By integrating data-driven learning with model-based filtering, the proposed framework effectively improves estimation stability and robustness. To evaluate the effectiveness of the proposed method, experiments were conducted under multiple operating conditions using the DST, US06, and FUDS datasets. The experimental results demonstrate that the proposed TCN-UKF method achieves superior SOC estimation accuracy compared with several benchmark methods, including CNN, GRU, LSTM, and standalone TCN models. Specifically, the proposed method improves the estimation accuracy by approximately 0.795%, 0.733%, 0.400%, and 0.187% compared with CNN, GRU, LSTM, and TCN, respectively. Furthermore, the MAE and RMSE are reduced to within 1.3% and 1.9%, indicating strong estimation performance under different operating conditions and initial states. Another advantage of the proposed framework is that it does not require explicit electrochemical modeling or complex parameter identification. Instead, it directly learns the nonlinear relationships between battery measurements and SOC through data-driven learning, while the UKF provides additional robustness against measurement noise and system uncertainty. Therefore, the proposed method offers a practical and efficient solution for SOC estimation in lithium-ion battery management systems.

**Further work** will further investigate several aspects to enhance the practical applicability of the proposed framework. First, the robustness of the model will be evaluated under a wider temperature range, particularly in extreme conditions below 0°C or above 40°C. Second, since real-world electric vehicle operation rarely involves complete charge–discharge cycles, future studies will focus on SOC estimation based on partial charge–discharge data and other health-related indicators. Third, to improve adaptability across different battery chemistries and battery types, transfer learning strategies will be explored. By transferring knowledge learned from one battery chemistry to another, the TCN-UKF framework can potentially reduce the amount of required training data and accelerate model deployment in practical battery management systems.
